# Clinical value of patient-specific three-dimensional printing of congenital heart disease: Quantitative and qualitative assessments

**DOI:** 10.1371/journal.pone.0194333

**Published:** 2018-03-21

**Authors:** Ivan Wen Wen Lau, Dongting Liu, Lei Xu, Zhanming Fan, Zhonghua Sun

**Affiliations:** 1 Department of Medical Radiation Sciences, Curtin University, Perth, Australia; 2 Department of Radiology, Beijing Anzhen Hospital, Capital Medical University, Beijing, China; Universita degli Studi Magna Graecia di Catanzaro, ITALY

## Abstract

**Objective:**

Current diagnostic assessment tools remain suboptimal in demonstrating complex morphology of congenital heart disease (CHD). This limitation has posed several challenges in preoperative planning, communication in medical practice, and medical education. This study aims to investigate the dimensional accuracy and the clinical value of 3D printed model of CHD in the above three areas.

**Methods:**

Using cardiac computed tomography angiography (CCTA) data, a patient-specific 3D model of a 20-month-old boy with double outlet right ventricle was printed in Tango Plus material. Pearson correlation coefficient was used to evaluate correlation of the quantitative measurements taken at analogous anatomical locations between the CCTA images pre- and post-3D printing. Qualitative analysis was conducted by distributing surveys to six health professionals (two radiologists, two cardiologists and two cardiac surgeons) and three medical academics to assess the clinical value of the 3D printed model in these three areas.

**Results:**

Excellent correlation (r = 0.99) was noted in the measurements between CCTA and 3D printed model, with a mean difference of 0.23 mm. Four out of six health professionals found the model to be useful in facilitating preoperative planning, while all of them thought that the model would be invaluable in enhancing patient-doctor communication. All three medical academics found the model to be helpful in teaching, and thought that the students will be able to learn the pathology quicker with better understanding.

**Conclusion:**

The complex cardiac anatomy can be accurately replicated in flexible material using 3D printing technology. 3D printed heart models could serve as an excellent tool in facilitating preoperative planning, communication in medical practice, and medical education, although further studies with inclusion of more clinical cases are needed.

## Introduction

Congenital heart disease (CHD) is the most common birth defect among the newborns [1]. Despite it being so common, patient management of CHD remains challenging due to its complexity and heterogeneity [2–5]. As the cardiovascular morphology varies greatly between individual patients, different surgical options and patient managements are required for each specific case [2]. For this reason, it is imperative to have thorough understanding of the spatial relationship between the intra-cardiac structures, in order to decide the best surgical options [2, 4, 6, 7].

To date, diagnostic assessment and pre-operative planning of CHD is based on volumetric data such as computed tomography (CT), magnetic resonance imaging (MRI), and echocardiography. In conjunction with the three-dimensional (3D) reconstruction method, 3D virtual model of human’s heart can be generated [3–6, 8]. Even though it allows the observers to visualize the heart from various angles, this method falls short in providing full comprehension of the intra-cardiac structures, as it still relies on interpretation from two-dimensional (2D) flat screen [3, 5, 6, 7–11].

In order to resolve this shortcoming, there have been several studies on fabrication of 3D printed cardiac models of CHD. These models were reported to facilitate the surgical decision-making and planning, enhance the patient-doctor communication, and improve the knowledge acquisition among the patients, students, and junior doctors [2–10, 12–30]. The ultimate reason for 3D printed models to outperform current diagnostic tools is because they can be tangibly manipulated and provide additional spatial information of the heart and surrounding cardiovascular structures [5, 6, 14, 19, 20, 26].

However, the evidence to suggest the dimensional accuracy of the 3D printed cardiac models is lacking. Most of the studies in current literature only investigate the clinical usefulness of the models without reporting their dimensional accuracy. Additionally, most of the current studies are case reports or case series focusing on the utility of 3D printed heart models in preoperative planning [2, 4–6, 12, 19–21, 23, 24, 26, 29–31]. There is limited evidence to suggest whether the health professionals and medical academics find the 3D printed heart model useful in their practice. Hence, a detailed investigation of the multi-directional use of the 3D printed heart model is required. Further, most of the models generated in the existing studies were solid, which could not resemble the softness or tissue property of human heart tissues [2–5, 7–9, 13–15, 18, 20, 22, 23, 26–29].

Thus, this study presents a preliminary experience in creating flexible 3D printed heart model from cardiac CT angiography (CCTA). The aim of this study was twofold: (1) to investigate the dimensional accuracy of the 3D printed heart model, and (2) to evaluate the clinical value and feasibility of flexible 3D printed models of CHD, in particular for preoperative planning, medical education, and communication in clinical practice.

## Materials and methods

### Selection of sample cases for image post-processing

Ethical approval was obtained from Curtin Human Research Ethics Committee. The ethics committee waived the requirement for informed consent due to the retrospective nature of the study and use of de-identified images. Five cases of de-identified CCTA images of complex CHD in Digital Imaging and Communications in Medicine (DICOM) format were obtained from a radiology archive. During the cases selection process, only cases with patients aged from 0–15 years old were included.

All CCTA was performed at 70 kV on a 256-slice GE Revolution scanner (Revolution CT, GE Healthcare, Waukesha, WI) or a 128-slice Siemens Definition Flash (Somatom Definition Flash, Siemens Healthcare, Forchheim, Germany). The contrast medium was administered according to the following protocol:

Injection rate: weight (kilograms) x 0.1 mL/s

Volume of contrast medium: injection rate x 20 s

To reduce the radiation dose to the paediatric patients, prospective electrocardiogram (ECG) triggering technology and tube current modulation were utilized. The selected cases were also ensured to have slice thickness of less than 0.75 mm to guarantee the resolution of acquired images for 3D printing purpose.

### Image segmentation and post-processing

**Fig 1:**
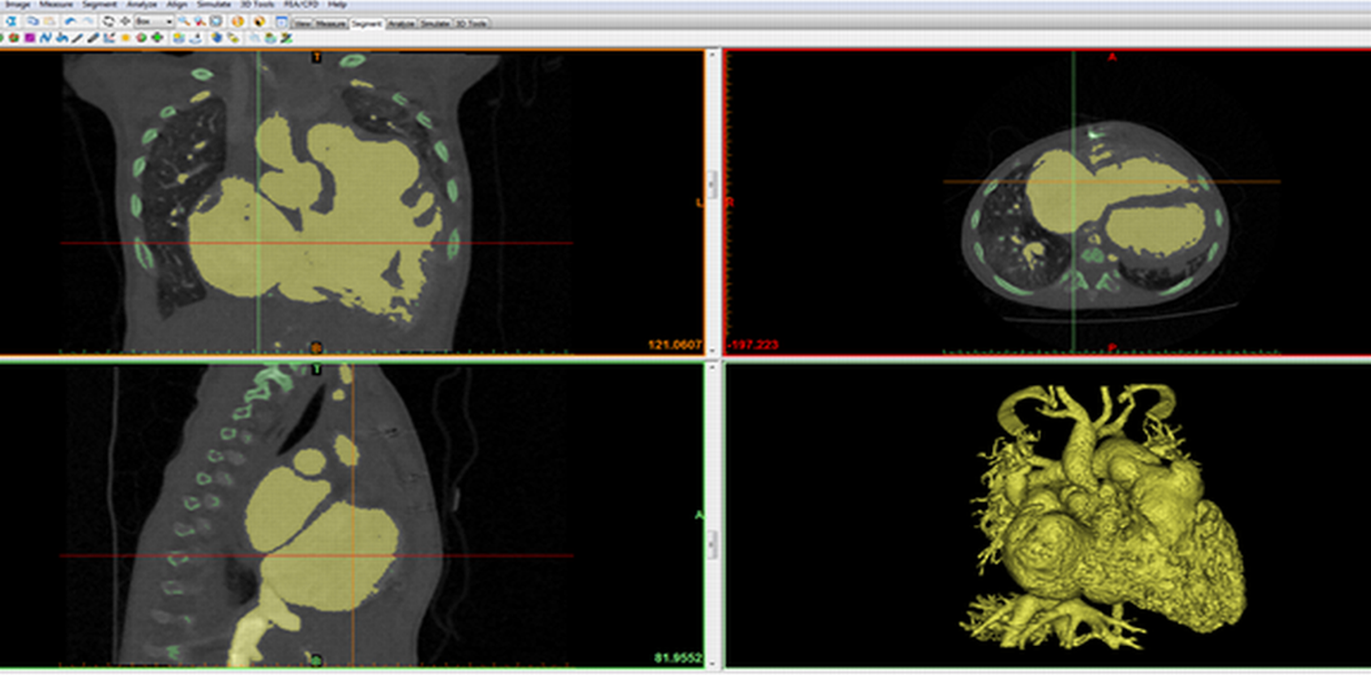
Image segmentation using *MIMICS Innovation Suite software*. Contrast-enhanced blood and cardiac chambers were highlighted and segmented from the surrounding soft tissue and bony structures.

All five cases were segmented using *Mimics Innovation Suite* software (Materialise HQ, Leuven, Belgium) using thresholding and region growing methods [32]. In the segmentation process, the blood pool in the heart was isolated from other anatomical structures (Fig 1). Following that, a layer of arbitrary thickness of 2 mm was added onto the blood pool surface to represent imaginary heart wall using *3-Matic* software (Materialise HQ, Leuven, Belgium). The digital model was hollowed and smoothed before being exported in Standard Tessellation Language (STL) format [32].

The STL file exported from *3-Matic* was loaded into *Geomagic Studio 12* (3D Systems, Inc. Korea) to separate the digital model into two compartments. This was done so that the intra-cardiac structures can be better demonstrated. The model was cut through a plane transecting right atrium and right ventricle to provide a viewing window for the ventricular septal defect (VSD). Mesh doctor function was also used to optimize the file for 3D printing.

The time to generate the STL model was limited to 2 hours to ensure the practicability of producing 3D printed heart models. Only one digital model that requires least manual edition but with excellent demonstration of cardiac anatomical structures and pathologies was chosen for 3D printing.

### Three-dimensional printing

The chosen digital model was printed with a commercial printer Stratasys (Objet Eden 260VS) using Polyjet printing technology in Tango Plus material, which is a rubber-like material that has more resemblance to human heart tissue when compared to hard plastic [33, 34]. After obtaining the physical 3D printed heart model, contrast-enhanced CT scan of the 3D model was performed to determine model’s accuracy. This was conducted on a 64-slice CT scanner (Philips Brilliance 64; Philips Medical Systems, Netherlands) using a contrast enhanced chest protocol at 120 kV, with a slice thickness of 0.6 mm. For the contrast scan, the model was submerged in a container filled with approximately 1L of fluid which contained 100 mL of Ultravist 300 (Bayer Australia Ltd, Pymble, NSW, Australia) and 900 mL of water. This allows for generation of CT attenuation of 250 HU which is similar to that used in routine CCTA.

### Quantitative measurements of anatomic accuracy

**Fig 2:**
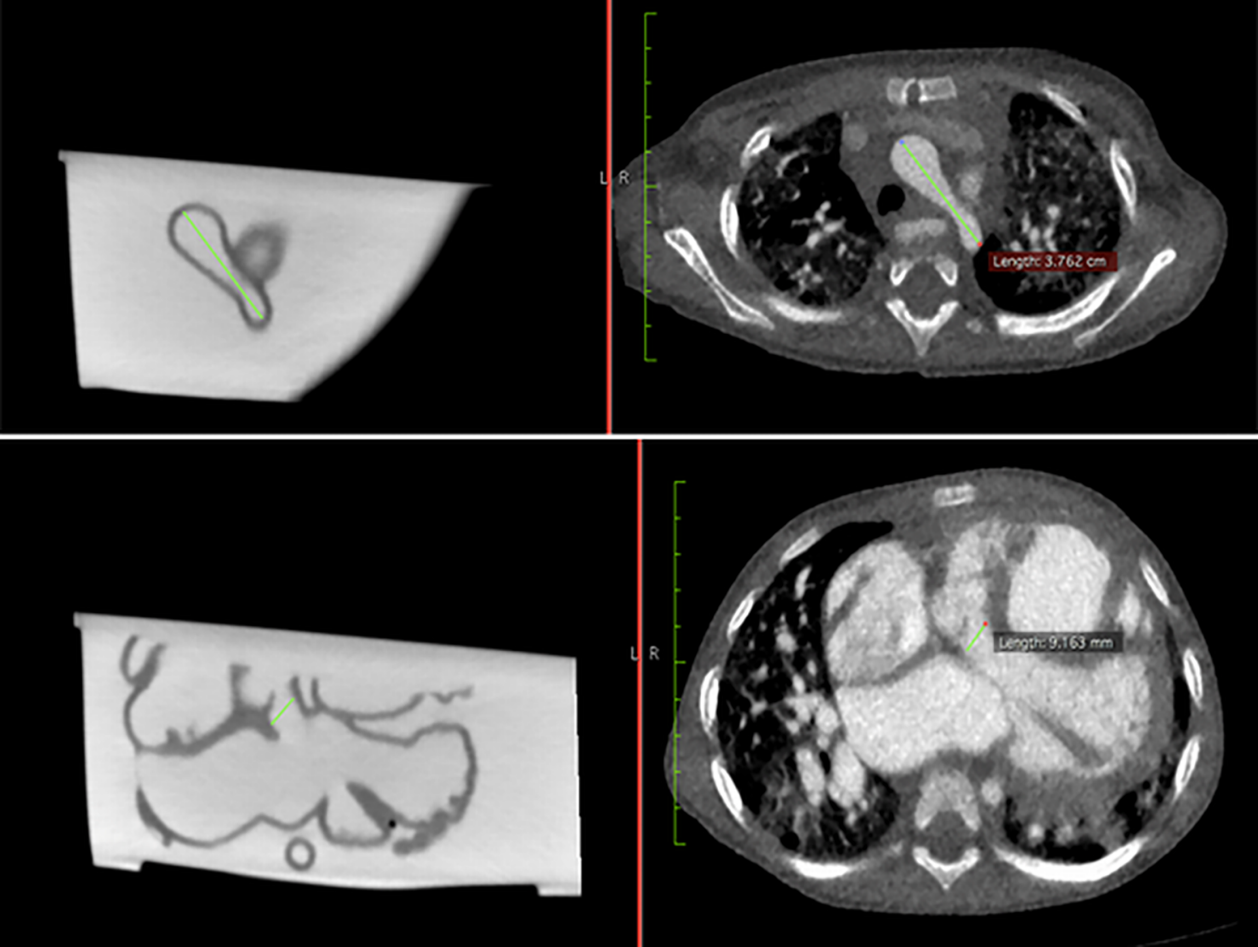
An example showing how measurements were taken at the aortic arch (top row) and ventricular septal defect (bottom row) using ruler function in *Horos*. The left column images refer to contrast-enhanced CT images of the 3D printed model, while the right column images indicate original CCTA images.

Measurements of different anatomical locations were taken at two stages for comparison: original CCTA and contrast-enhanced CT images of the 3D printed model. Both stages of measurements were done using the ruler function in *Horos* (Horos Project), which is an open-source DICOM viewer (Fig 2). The internal diameter of ten anatomical locations were measured and compared. These anatomical locations consisted of the base of brachiocephalic trunk, aortic arch, ascending aorta, left pulmonary artery, right pulmonary artery, pulmonary trunk, descending aorta, VSD, right atrium and left ventricle. In order to reduce bias in results, the measurement for each anatomical location was repeated for three times by two independent observers.

### Qualitative assessment of clinical value

To assess the clinical value of the 3D printed model, interviews and surveys were conducted with six health professionals consisting of two radiologists (S1 File), two cardiologists (S2 File), and two cardiac surgeons (S3 File), as well as three medical academic staff (S4 File) who voluntarily participated in the survey (. The health professionals were from major public and private practices, whereas the medical academic staffs were recruited from Curtin University.

Each participant had access to the de-identified CCTA dataset as well as the 3D printed model either online or physically. A brief introduction of the 3D printed model was given to the participants before they were asked to compare the CCTA and 3D model. For the participants to have online access of the 3D printed heart model, an introductory video of it was uploaded to social media. The de-identified CCTA dataset was also uploaded to DICOM Library. DICOM Library is an online platform to share DICOM file for educational and scientific purposes [35].

The participants were then asked to complete a set of questionnaires. The questions focused on evaluating their perception of 3D printing of CHD in preoperative planning, medical education, and communication in clinical practice, by using 3-point Likert scale and written response format. Each group of professionals received a different set of questionnaires, as the questionnaires were specifically designed to be relevant to the participants’ scope of practice. The questionnaires were generated and modified based on the findings of previous studies [4, 6, 7, 9, 14–16, 28–30, 33, 34].

### Statistical analysis

Due to the limited number of case and participants, the data analysis of this study was strictly limited to descriptive statistics. For quantitative analysis, Pearson’s correlation coefficient was used to assess correlation of the measurements on original CCTA and 3D printed model [27]. All the measurements were recorded in Microsoft Excel with the mean values, mean difference, and correlation coefficient calculated. For qualitative analysis, the questions were grouped into three main categories, namely preoperative planning, communication in clinical practice, and medical education. The responses of each category were entered into SPSS 24.0 (SPSS, IBM Corporation, Armonk, NY, USA) and tabulated in frequency tables.

## Results

### Image post-processing and three-dimensional printing

**Fig 3:**
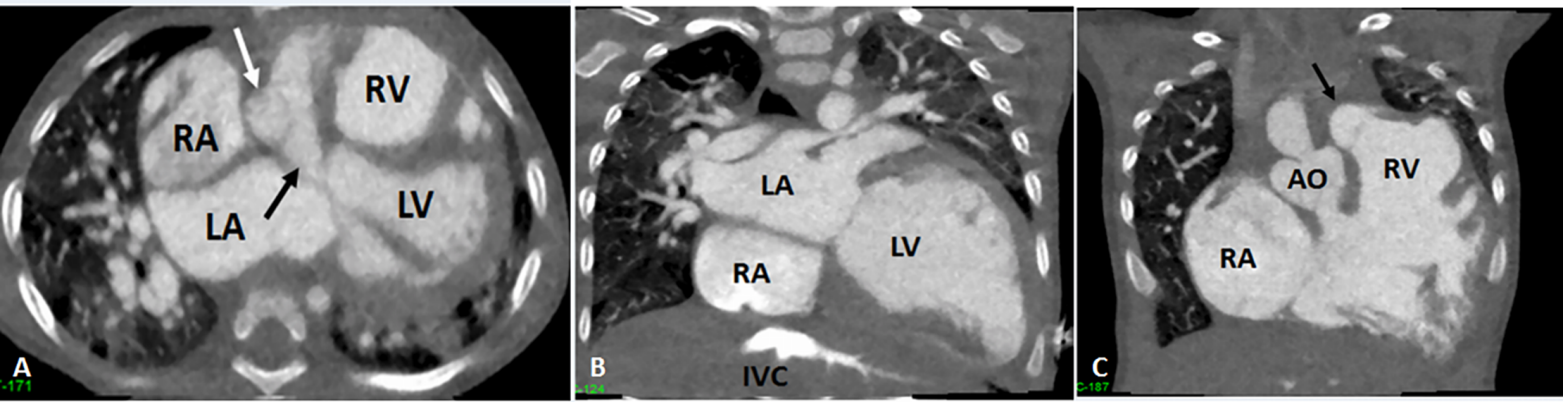
Cardiac computed tomography (CT) angiography showing double outlet right ventricle and ventricular septal defect (VSD). A: 2D axial CT image showing the four-chamber view with sub-aortic VSD (black arrow). B: 2D coronal CT image showing left atrium (LA), right atrium (RA) and left ventricle (LV). C: 2D coronal CT image showing that aorta (AO) and pulmonary artery (black arrows) all arise from right ventricle (RV). IVC-inferior vena cava, white arrow in A refers to ascending aorta.

The chosen case for 3D printing is a case of a 20-month-old boy who was diagnosed with double outlet right ventricle with a sub-aortic VSD. The CCTA demonstrated both the aorta and pulmonary trunk originate from the right ventricle. Both the great arteries are transposed with aorta at the right side. The aortic arch and descending aorta are narrowed. The VSD is located below the aortic valve, with the size of approximately 13.2 mm (Fig 3).

**Fig 4:**
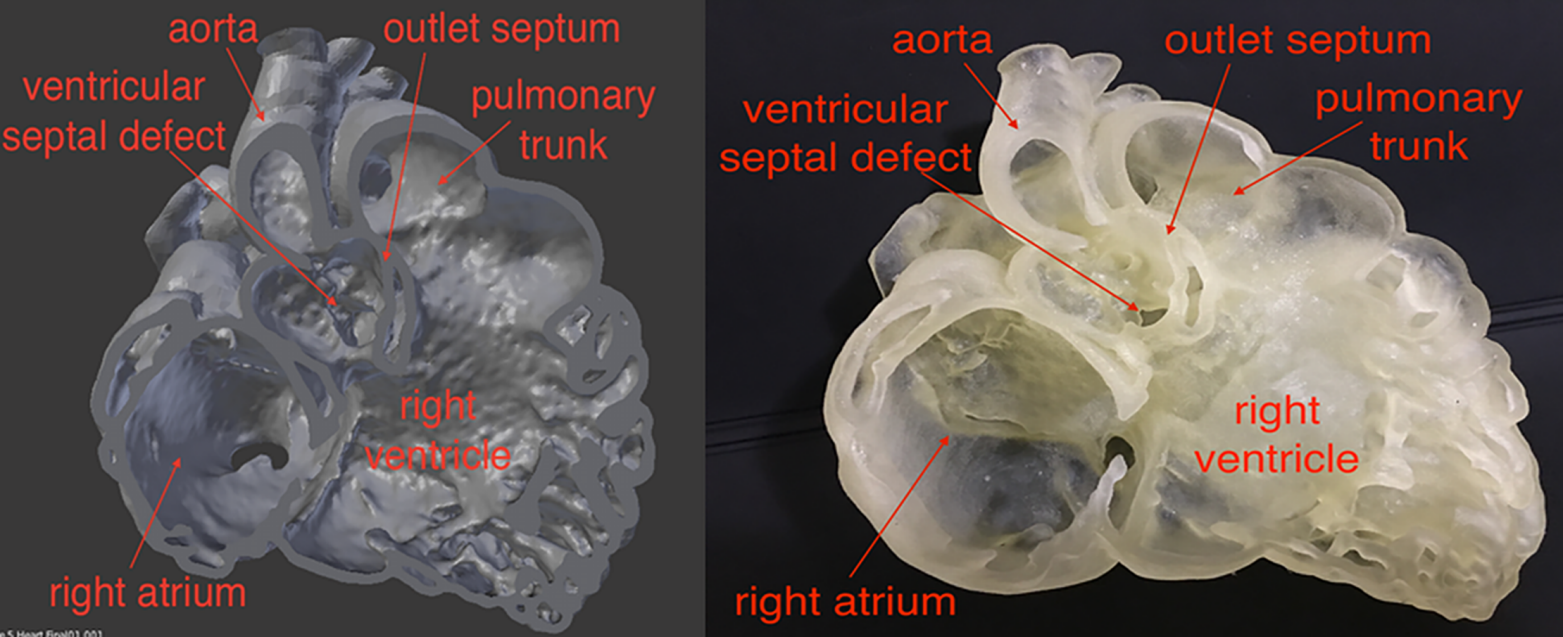
Labeled screen-display of the virtual model (left) and photograph of the 3D printed heart model (right). Reprinted with permission under the open access from Lau and Sun [31].

**Fig 5:**
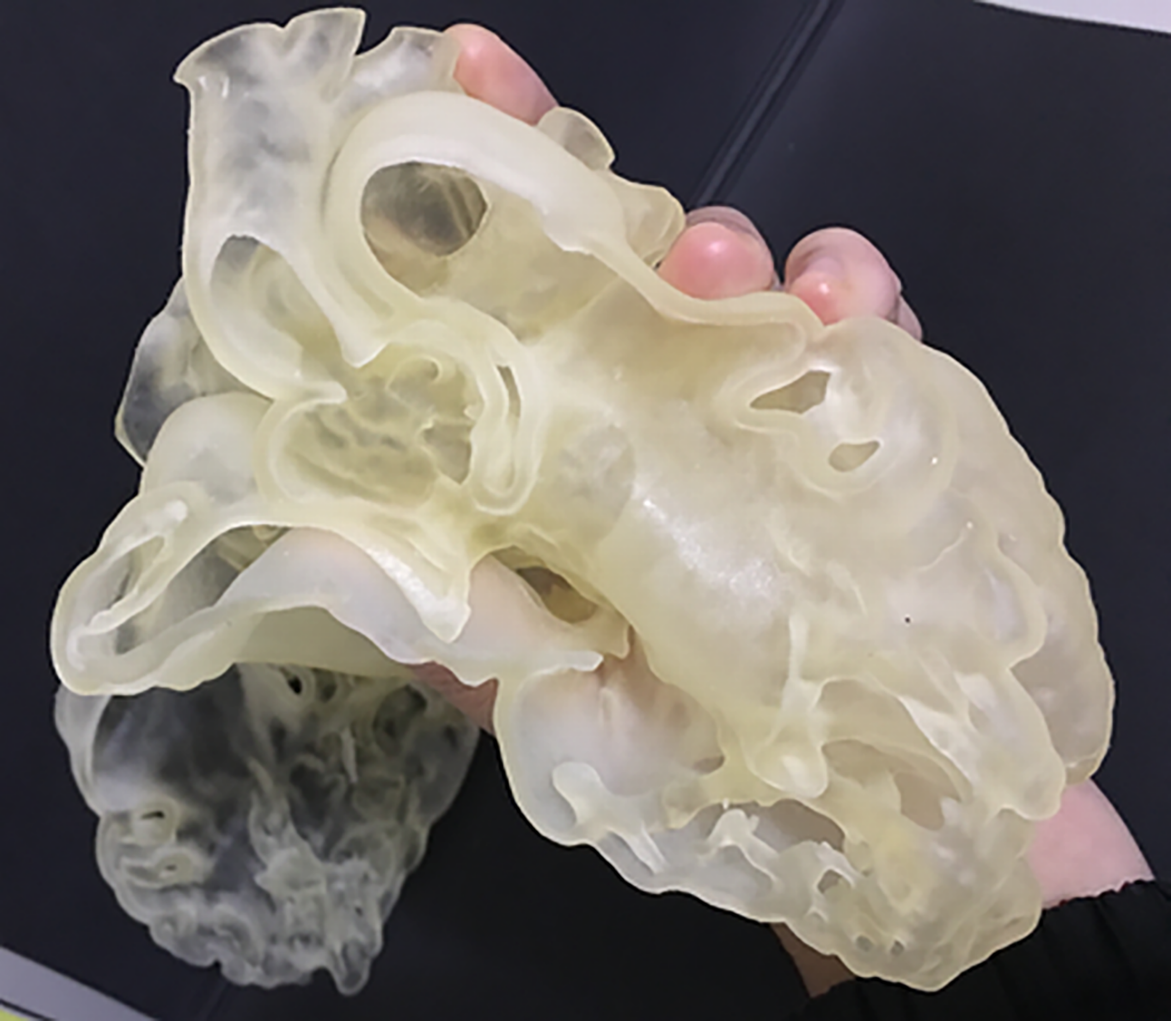
Demonstration of the flexibility of the 3D printed heart model in Tango Plus material. Reprinted with permission under the open access from Lau and Sun [31].

The time taken to complete the image post-processing using Mimics was 15 minutes. The total cost of 3D printing was around AUD $300. The resulting 3D model shows that it is feasible to replicate the heart in rubber-like material, with excellent demonstration of intra-cardiac structures (Figs 4 and 5).

### Quantitative measurements of anatomical accuracy

**Fig 6:**
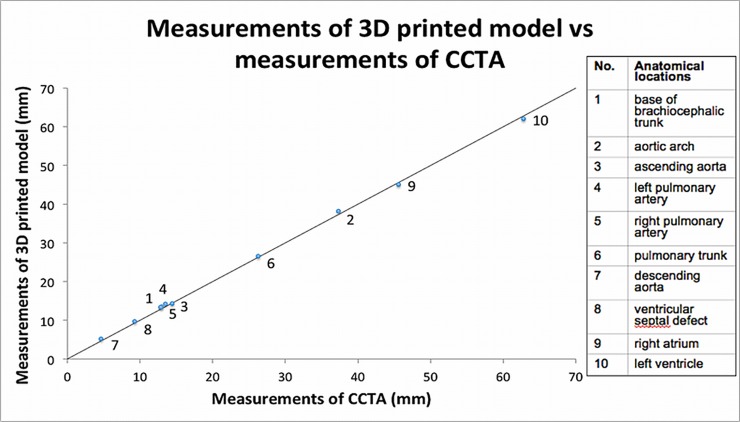
Scatter plot of measurements of 3D printed model against measurements of cardiac computed tomography angiography (CCTA). Each data point is assigned with a number to represent different anatomical locations. 3D: three-dimensional.

A strong correlation (r = 0.99) was demonstrated between the CCTA and 3D printed model measurements, indicating that the 3D printed model generated in this study is highly accurate in replicating anatomical cardiac structures. Overall, the 3D printed model marginally over-estimated the size of the heart when compared to the CCTA measurements, but only by 0.23 mm in average. Fig 6 demonstrates a scatter plot comparing the measurements of 3D printed model and CCTA. Note that all the data points lie very closely with the perfect correlation line (r = 1.00).

### Qualitative assessment of clinical value

Overall, all the participants have at least three years of working experience in the corresponding professional field. Eight out of nine participants had encountered challenges in their profession before due to limitations of current visualization techniques, whereas six out of nine participants reported that they had used 3D printed cardiac models in their practice previously. The average satisfaction score of the model was 8.4 out of 10.

### Preoperative planning in congenital heart disease

Four out of six health professionals indicated that the 3D printed models are useful in planning interventions, while three out of six reported that it could be useful in testing devices for pre-surgical simulation (Table 1). There were no participants who responded ‘no’ to the questions in this category, implying that all the participants to some extent agreed that the 3D printed model is useful in pre-surgical planning.

**Table 1:** Frequency table of usefulness of three-dimensional printed heart models in preoperative planning.

	Do you think patient-specific 3D models are helpful in…	
Count %	planning interventions?	testing device for pre-surgical simulation?
Yes	4 (66.7%)	3 (50.0%)
Maybe	2 (33.3%)	3 (50.0%)
No	0 (0.0%)	0 (0.0%)
Total^*^	6 (100.0%)	6 (100.0%)

*with cardiac surgeons, cardiologists, and radiologists as participants

3D: three-dimensional

Both the cardiac surgeons agreed that the 3D printed models would be able to provide additional information of the pathology compared to conventional imaging and computer simulation. They reported that the 3D printed cardiac model is helpful for them to appreciate potential procedural difficulties, and hence will be able to increase the success rate of the surgery.

### Communication in clinical practice

The two cardiac surgeons and two cardiologists were asked whether the 3D printed model is useful in enhancing patient-doctor communication, and all of them reported that the model would be invaluable in improving the consultation experience as they would be able to explain the CHD to the patients more efficiently. They also thought that the patients could understand the condition of their heart better with the use of 3D printed model. However, when being asked whether the consultation time could be reduced, only two of them responded ‘yes’ (Table 2).

**Table 2:** Frequency table of usefulness of three-dimensional printed heart models in communication in medical practice.

	Do you think…			
Count %	you can clarify/describe the pathology to the patients/health professionals better using this model, rather than using the DICOM dataset itself?	the patients’/parental understanding of the disease and surgical procedures will be enhanced with the use of 3D printed model during consultation time?	the model can improve the consultation experience?	the model can shorten the consultation time?
Yes	4 (100.0%)	4 (100.0%)	4 (100.0%)	2 (50.0%)
Maybe	0 (0.0%)	0 (0.0%)	0 (0.0%)	1 (25.0%)
No	0 (0.0%)	0 (0.0%)	0 (0.0%)	1 (25.0%)
Total^*^	4 (100.0%)	4 (100.0%)	4 (100.0%)	4 (100.0%)

* with cardiac surgeons and cardiologists as participants

DICOM: Digital Imaging and Communications in Medicine

3D: three-dimensional

**Fig 7:**
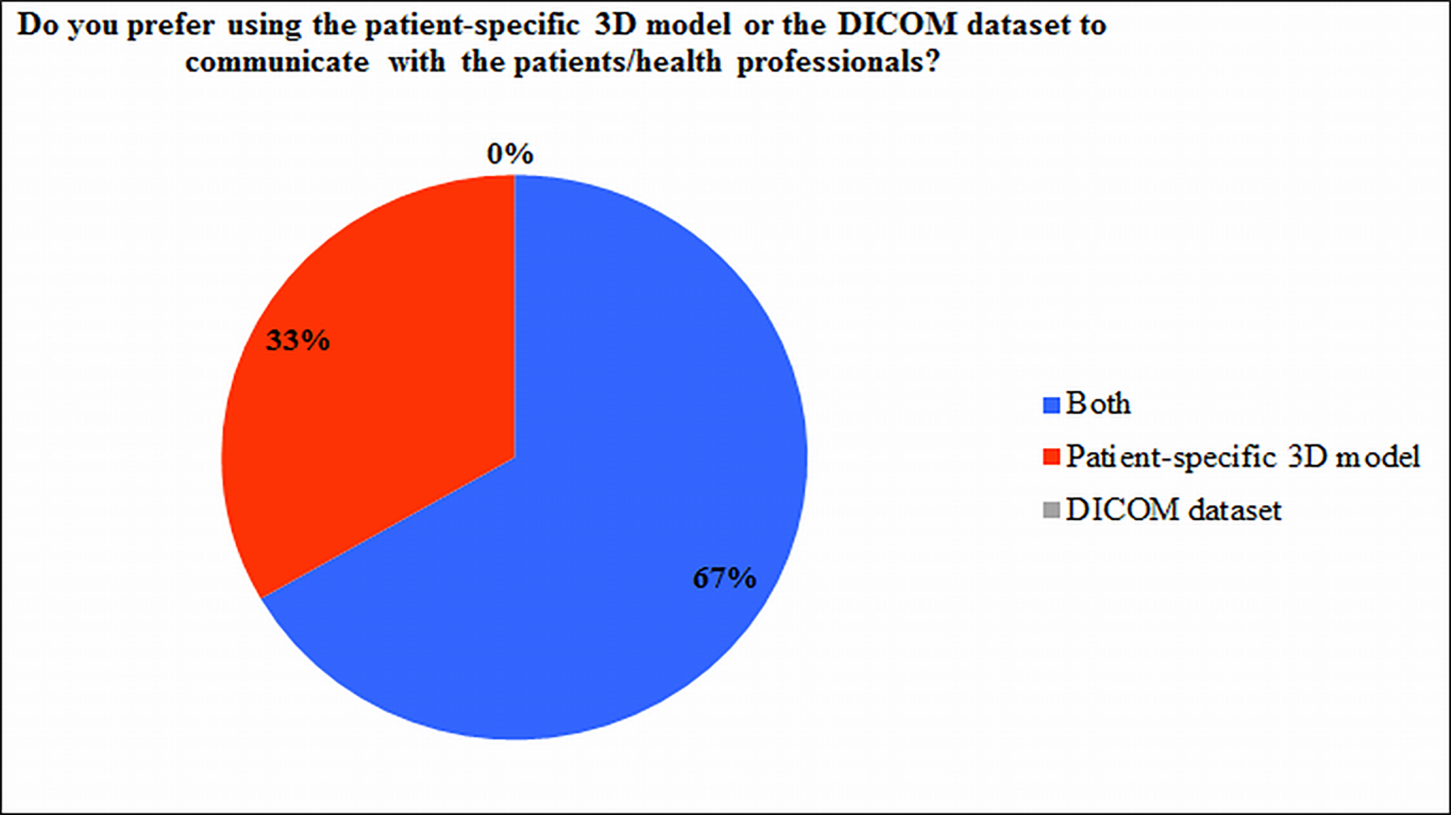
Chart representation of the responses obtained from cardiologists, cardiac surgeons and radiologists. 3D: three-dimensional; DICOM: Digital Imaging and Communications in Medicine.

Interestingly, when all the health professionals were asked whether they prefer to use 3D printed model or medical images to communicate with the patients or other health professionals, four out of six indicated they prefer to use both of them as a medium in communication (Fig 7).

### Medical education

The three medical academic staff members were asked about their opinion of using 3D printed cardiac models in teaching anatomy and pathology. All of them found it invaluable, and indicated that the students will be able to learn the pathology quicker with better understanding. However, two medical academics implied that the 3D printed model generated in this study could not help the students to learn normal heart anatomy (Table 3). This is because the printed model is a diseased heart, so it could not represent the normal cardiac structures. Hence, they suggested that a ‘normal’ 3D printed cardiac model should be used for comparison when teaching the students.

**Table 3:** Frequency table of usefulness of three-dimensional printed heart models in medical education.

	Do you think this model…		
Count %	can enhance the students’ knowledge of normal heart anatomy?	can enhance the students’ knowledge of cardiac pathology?	will enable the students to learn the disease quicker?
Yes	1 (33.3%)	3 (100.0%)	3 (100.0%)
Maybe	0 (0.0%)	0 (0.0%)	0 (0.0%)
No	2 (66.7%)	0 (0.0%)	0 (0.0%)
Total^*^	3 (100.0%)	3 (100.0%)	3 (100.0%)

* with medical academics as participants

## Discussion

3D printing in medicine was initially introduced in maxillofacial and orthopedic specialties, mainly for preoperative simulation. It was only in recent years that this technique has expanded to cardiovascular diseases. Its clinical value in this area is promising, although more research is needed [4, 6, 26, 29]. A number of studies have shown encouraging results with the use of 3D printed heart models with CHD in preoperative planning, medical education, and communication in clinical practice [2–10, 12–30]. This study further validates these findings by conducting both quantitative and qualitative assessments on a realistic 3D printed heart model with CHD.

Only a few existing studies reported on the dimensional accuracy of the 3D printed heart models. Despite so, all of them are in general agreement that the 3D printed heart models are accurate, with the reported mean difference ranges from 0.05 ± 0.17 mm to 0.4 ± 0.9 mm [7, 8, 26, 27, 30]. There were also a few studies attempted to produce flexible 3D printed model showing that the flexibility of the material allows the surgeons to rehearse the surgical procedures and thus improve the surgical outcomes [6, 12, 24, 30]. Previous studies have also validated the ability of the 3D printed heart models to facilitate decision-making of treatment plan, as it enhances the perception of spatial relationships between the cardiac structures [2, 4–7, 12, 14, 21, 23, 24, 26, 29, 30]. However, studies pointed out that the 3D printed heart models should only be used to complement the current diagnostic tools, instead of stand-alone tool for preoperative planning [4, 18, 20, 26].

The preliminary experience in this study demonstrates that: (1) it is feasible to accurately replicate a heart in flexible material; and (2) the 3D printed heart model of CHD is useful in pre-operative planning, medical education, and communication in medical practice. These positive findings are significant as this is the first report to investigate the accuracy and the multi-directional utility of the 3D printed heart model in a holistic approach. To our knowledge, this is also the first time to confirm model accuracy by performing contrast-enhanced CT scan of the 3D printed heart model for measurement of the intra-cardiac structures (Fig 2), since other studies used caliper for measurements [26, 30]. This has significant clinical value because the luminal size of the flexible heart model changes when a caliper is placed within it. Thus, the approach used in this study to measure the 3D printed model to ensure its size is in its original state, which is demonstrated through performing CT scan on the 3D printed model.

The mean difference of 0.23 mm between the measurements of the 3D printed heart model and the original CCTA could be caused by several reasons. First, it is possible that the measured locations on both CT datasets were not perfectly at the same point. Even though the CT scan of the 3D printed model was reconstructed manually to match the heart orientation of the original CCTA, there is a chance to have slight variations between the analogous locations, impeding a perfect comparison. Second, the image resolution and noise of the source data could also introduce errors in defining the boundaries of the anatomical structures [36]. Although the STL file was smoothed to reduce artifacts caused by the noise prior to printing, the smoothing factor used in this study might not be suitable. Third, the chosen threshold value during the segmentation process might be too low, as the 3D printed model marginally over-estimated the heart size. Fourth, the resolution limits of the 3D printer could also have contributed to the variance between the 3D printed heart model and original CCTA [36]. This mean difference however, is considered acceptable, as it falls within the range of the mean differences reported by other studies [7, 8, 26, 27, 30].

Overall, the cardiac surgeons and cardiologists responded positively with respect to the use of 3D printed heart model in communication within medical practice. However, not all of them agreed that the duration of consultation could be reduced with the use of 3D printed heart model. In a study carried out by Biglino et al, it was found out that the consultation involving the use of 3D printed model lasted 5 minutes longer than usual [13]. This might not necessarily be a negative finding, as the use of 3D printed model could lead to more detailed discussion between the patients and the doctors, thus allowing the patients to achieve better understanding of their condition [13]. Additionally, most of the health professionals prefer to use both medical images and 3D printed heart models in communication. This might indicate that the 3D printed models could not entirely replace the existing approach in communication, but rather to serve as a complementary tool in patient-doctor communication. It could also be due to the fact that the health professionals still rely on 2D medical images for communication, as it is how they have been trained and practicing, although this needs to be further investigated [14]. Some participants also indicated that the current 3D printed model could be improved by color-coding the venous and arterial blood, in order to enhance the patients’ and students’ understanding.

This study has several limitations. First, although comparing the measurements of the original CCTA and 3D printed heart model at ten different anatomical locations was an indicator of the 3D model’s accuracy, it is worthwhile to note that the results may not necessarily reflect the accuracy of the entire model, as the measurements were only taken in axial plane (Fig 2). It is not justified whether 3D printing could distort the size of the heart in other planes, and whether this difference in measurement is uniform in all three dimensions. Future studies are suggested to measure the accuracy of 3D printed models in all three dimensions, including axial, coronal, and sagittal planes to assess the accuracy of the entire model.

Second, there is only one 3D printed heart model generated in this study, therefore, the results lack generalization. Despite this limitation, findings in this study are consistent with those reported in a multi-centre study representing a larger group of patients with 3D printing of CHD [37]. In their recent report involving 10 international centres with inclusion of 40 cases, Valverde et al investigated the impact of 3D printed models on surgical planning and treatment of CHD. Forty 3D printed heart models were generated using cardiac CT and MRI data. Excellent correlation was found in vascular measurements between 3D printed models and original CT or MRI images, with a mean bias of -0.27 ± 0.73 mm, which is very similar to our findings. Further, 3D printed models was ranked satisfactory (>9.0 out of 10) by surgeons and paediatric cardiologists. In nearly half of the cases, 3D models were found to help define the surgical approach [37]. Third, only high quality dataset was used as the source data, therefore the recorded duration for segmentation could not reflect the actual segmentation time for low quality images. In real clinical practice, it is difficult to obtain perfectly CCTA, thus it will require more effort and time in segmentation [6]. Fourth, the 3D printed model generated in this study is static, so it does not allow the observers to understand the haemodynamic function of the heart [2, 11].

As 3D printing in cardiovascular specialty is still in its infancy, future studies should include more cases and participants to confirm the dimensional accuracy and clinical value of the 3D printed heart models. It will also be worthwhile to include surveys with the parents or patients with CHD, as they are the stakeholders who would benefit directly from this novel technology. Additionally, future studies should focus on developing standardized method in generating 3D printed heart models. In the current literature, there is a great variation in terms of how 3D printed models were fabricated, including the source data and segmentation method [18, 38, 39]. These variations can possibly affect the accuracy of the 3D printed models [2, 37]. Furthermore, future investigations should also focus on analyzing the cost-benefit of 3D printing of CHD [3, 9, 13, 18]. The cost of 3D printing was considered as one of the main factors to impede the wide application of 3D printing in medicine [9, 33, 40]. There is a need of a comprehensive cost-benefit analysis to determine the practicability of 3D printing of CHD.

In conclusion, this study has preliminarily assessed the dimensional accuracy as well as the clinical value of flexible patient-specific 3D printed heart models. The flexible 3D printed heart model can be accurately reproduced from cardiac CT images. It is found to facilitate preoperative planning and improve the surgical outcome; enhance patient-doctor and inter-professional communication; and improve approach in medical teaching. These findings warrant further investigations in 3D printing of congenital heart disease.

## Supporting information

S1 File Survey questionnaire for two radiologists. (DOCX)

S2 File Survey questionnaire for two cardiologists. (DOCX)

S3 File Survey questionnaire for two cardiac surgeons. (DOCX)

S4 File Survey questionnaire for three medical academics. (DOCX)
