# Supplementary material for: Clinical value of patient-specific three-dimensional printing of congenital heart disease: Quantitative and qualitative assessments
Source: PLoS One. 2018 Mar 21;13(3):e0194333. doi: 10.1371/journal.pone.0194333 (PMC5862481; doi:10.1371/journal.pone.0194333)
Supplement: S1 File — (DOCX) [file pone.0194333.s001.docx]

**Questionnaire for radiologists**

**General details**

1) For how many years have you performed practice in your area of expertise?

☐ <3 years ☐ 3-8 years ☐ >8 years

2) Have you had any experience that the CHD is too complex where accurate diagnosis could not be made, or, communication with other health professionals regarding the pathology was unsuccessful? If yes, please briefly describe the experience:

3) Have you previously used a 3D model as a medium to explain the pathology to other health professionals?

☐ Yes ☐ No ☐ Maybe

**Degree of verisimilitude of the 3D model**

4) Does this model accurately display the cardiac structures as portrayed by the CT dataset?

☐ Yes ☐ No ☐ Maybe

5) Do you think your understanding of the disease and surgical procedures will be enhanced with the use of 3D model?

☐ Yes ☐ No ☐ Maybe

**Usefulness of the model as pre-operative planning tools**

6) Do you think patient-specific 3D printed models are helpful in planning interventions?

☐ Yes ☐ No ☐ Maybe

7) Do you think patient-specific 3D printed models are helpful in testing devices for pre-surgical simulation and selecting appropriate equipment and devices for use?

☐ Yes ☐ No ☐ Maybe

8) Do you think patient-specific 3D printed models are helpful in intra-operative orientation?

☐ Yes ☐ No ☐ Maybe

If yes, do you think it can reduce operative time?

☐ Yes ☐ No ☐ Maybe

**Usefulness of the model as a medium to communicate in medical practice**

9) Do you think you would be able to clarify/describe the pathology to the patients/health professionals better using this model, rather than using the DICOM dataset itself?

☐ Yes ☐ No ☐ Maybe

10) Do you think the communication between the health professionals will be more effective with the use of 3D printed model?

☐ Yes ☐ No ☐ Maybe

11) If you were to choose, do you prefer using the patient-specific 3D model or the DICOM dataset to communicate with the patients/health professionals?

☐ Patient-specific 3D model ☐ DICOM dataset ☐ Both

**Applications and limitations**

12) From rank 1-5, with 1 being the most relevant, please rank the most relevant potential applications of 3D printed model.

[ ] Preoperative planning

[ ] Pre-surgical simulation

[ ] Intra-operative orientation

[ ] Communication in medical practice

[ ] Medical education

13) Which areas do you think that this model has to be improved in order to bring (more) benefits in medical field?

14) Do you think 3D printing of complex CHD is practical and feasible in the medical field?

☐ Yes ☐ No ☐ Maybe

**Overall Satisfaction with the 3D models**

15) How would you rank your overall satisfaction with the 3D model, from 1-10, with 10 being very satisfied. [ ]

16) Would you recommend 3D printing to your colleagues?

☐ Yes ☐ No ☐ Maybe

17) Have you got any other comments?
