# Supplementary material for: Clinical value of patient-specific three-dimensional printing of congenital heart disease: Quantitative and qualitative assessments
Source: PLoS One. 2018 Mar 21;13(3):e0194333. doi: 10.1371/journal.pone.0194333 (PMC5862481; doi:10.1371/journal.pone.0194333)
Supplement: S2 File — (DOCX) [file pone.0194333.s002.docx]

**Questionnaire for cardiologists**

**General details**

1) For how many years have you performed practice in your area of expertise?

☐ <3 years ☐ 3-8 years ☐ >8 years

2) Have you had any experience that the CHD is too complex and the patient management was made difficult, or the communication with patients during consultation time was unsuccessful? If yes, please briefly describe the experience:

3) Have you previously used a 3D model as a medium to communicate with patients during consultation?

☐ Yes ☐ No ☐ Maybe

**Degree of verisimilitude of the 3D model**

4) Does this model accurately display the cardiac structures as portrayed by the CT dataset?

☐ Yes ☐ No ☐ Maybe

**Usefulness of the model as a medium to communicate during consultation**

5) Do you think you would be able to clarify/describe the pathology and surgical procedures to the patients better using this model, rather than using the DICOM dataset itself?

☐ Yes ☐ No ☐ Maybe

6) Do you think the patients’/parental understanding of the disease and surgical procedures will be enhanced with the use of 3D model during consultation time?

☐ Yes ☐ No ☐ Maybe

7) Do you think the model can improve the consultation experience?

☐ Yes ☐ No ☐ Maybe

8) Do you think the model can shorten the consultation time?

☐ Yes ☐ No ☐ Maybe

9) If you were to choose, do you prefer using the patient-specific 3D model or the DICOM dataset to communicate with the patients?

☐ Patient-specific 3D model ☐ DICOM dataset ☐ Both

10) What other positive or negative impact(s) that you can think of with the use of 3D model during consultation?

**Other application of the model**

11) Do you think patient-specific 3D printed models are helpful in planning interventions?

☐ Yes ☐ No ☐ Maybe

12) Do you think patient-specific 3D printed models are helpful in testing devices for pre-surgical simulation?

☐ Yes ☐ No ☐ Maybe

13) Do you think patient-specific 3D printed models are helpful in intra-operative orientation?

☐ Yes ☐ No ☐ Maybe

14) From rank 1-5, with 1 being the most relevant, please rank the most relevant potential applications of 3D printed model.

[ ] Preoperative planning

[ ] Pre-surgical simulation

[ ] Intra-operative orientation

[ ] Communication in medical practice

[ ] Medical education

**Limitations and Feasibility**

15) Which areas do you think that this model has to be improved in order to bring (more) benefits in medical field?

16) Do you think 3D printing of complex CHD is practical and feasible in the medical field?

☐ Yes ☐ No ☐ Maybe

**Overall Satisfaction with the 3D models**

17) How would you rank your overall satisfaction with the 3D model, from 1-10, with 10 being very satisfied. [ ]

18) Would you recommend 3D printing to your colleagues?

☐ Yes ☐ No ☐ Maybe

19) Have you got any other comments? (This will be audio-recorded)
