# Supplementary material for: Clinical value of patient-specific three-dimensional printing of congenital heart disease: Quantitative and qualitative assessments
Source: PLoS One. 2018 Mar 21;13(3):e0194333. doi: 10.1371/journal.pone.0194333 (PMC5862481; doi:10.1371/journal.pone.0194333)
Supplement: S3 File — (DOCX) [file pone.0194333.s003.docx]

**Questionnaire for cardiac surgeons**

**General details**

1) For how many years have you performed practice in your area of expertise?

☐ <3 years ☐ 3-8 years ☐ >8 years

2) Have you had any experience that the CHD is too complex and the pre-surgical planning was made difficult, or the communication with patients during consultation time was unsuccessful? If yes, please briefly describe the experience:

3) Have you previously used a 3D model as a medium to plan interventions?

☐ Yes ☐ No ☐ Maybe

**Degree of verisimilitude of the 3D model**

4) Does this model accurately display the cardiac structures as portrayed by the CT dataset?

☐ Yes ☐ No ☐ Maybe

**Usefulness of the model as pre-operative planning tools**

5) Do you think patient-specific 3D printed models are helpful in planning interventions?

☐ Yes ☐ No ☐ Maybe

6) Do you think patient-specific 3D printed models are helpful in testing devices for pre-surgical simulation and selecting appropriate equipment and devices for use?

☐ Yes ☐ No ☐ Maybe

7) Do you think the 3D printed model is helpful for you to appreciate potential procedural difficulties?

☐ Yes ☐ No ☐ Maybe

8) Do you think the 3D printed model will help you to assess the likelihood of success or failure of the surgery? If yes, do you think the success rate of the surgery can be increased with the use of 3D printed model?

☐ Yes ☐ No ☐ Maybe

9) Does 3D printing add value in addition to conventional imaging and computer simulation during the process of pre-surgical planning?

☐ Yes ☐ No ☐ Maybe

10) Do you think patient-specific 3D printed models are helpful in intra-operative orientation? If yes, do you think it can reduce operative time?

☐ Yes ☐ No ☐ Maybe

**Usefulness of the model as a medium to communicate during consultation**

11) Do you think you would be able to clarify/describe the pathology and surgical procedures to the patients better using this model, rather than using the DICOM dataset itself?

☐ Yes ☐ No ☐ Maybe

12) Do you think the patients’/parental understanding of the disease and surgical procedures will be enhanced with the use of 3D model during consultation time?

☐ Yes ☐ No ☐ Maybe

13) Do you think the model can improve the consultation experience?

☐ Yes ☐ No ☐ Maybe

14) Do you think the model can shorten the consultation time?

☐ Yes ☐ No ☐ Maybe

15) If you were to choose, do you prefer using the patient-specific 3D model or the DICOM dataset to communicate with the patients?

☐ Patient-specific 3D model ☐ DICOM dataset ☐ Both

16) What other positive or negative impact(s) that you can think of with the use of 3D model during consultation?

**Other application of the model**

17) From rank 1-5, with 1 being the most relevant, please rank the most relevant potential applications of 3D printed model.

[ ] Preoperative planning

[ ] Pre-surgical simulation

[ ] Intra-operative orientation

[ ] Communication in medical practice

[ ] Medical education

**Limitations and Feasibility**

18) Which areas do you think that this model has to be improved in order to bring (more) benefits in medical field?

19) Do you think 3D printing of complex CHD is practical and feasible in the medical field?

☐ Yes ☐ No ☐ Maybe

**Overall Satisfaction with the 3D models**

20) How would you rank your overall satisfaction with the 3D model, from 1-10, with 10 being very satisfied. [ ]

21) Would you recommend 3D printing to your colleagues?

☐ Yes ☐ No ☐ Maybe

22) Have you got any other comments? (This will be audio-recorded)
