# Supplementary material for: Clinical value of patient-specific three-dimensional printing of congenital heart disease: Quantitative and qualitative assessments
Source: PLoS One. 2018 Mar 21;13(3):e0194333. doi: 10.1371/journal.pone.0194333 (PMC5862481; doi:10.1371/journal.pone.0194333)
Supplement: S4 File — (DOCX) [file pone.0194333.s004.docx]

**Questionnaire for medical lecturers and tutors**

|  | I have received information regarding this research and had an opportunity to ask questions. I believe I understand the purpose, extent and possible risks of my involvement in this project and I voluntarily consent to take part. |
| --- | --- |

**General teaching Details**

1) For how many years have you worked as an academic staff teaching medical anatomy and pathology units?

☐ <3 years ☐ 3-8 years ☐ >8 years

2) What is the current approach you use to teach anatomy and pathology units? (tick all that apply)

☐ cadaveric materials ☐ drawn diagrams ☐ pathological specimens
☐ 2D medical images ☐ 3D printed model

3) Did you have any challenges or barriers that you face when you are teaching anatomy classes? What are they?

**Degree of verisimilitude of the 3D models**

4) Does this model provide a more realistic visualization of the cardiac structures when compared to visualizing it from computer/cadaveric materials?

☐ Yes ☐ No ☐ Unsure

**Usefulness of the models as tools for medical education**

5) Do you think this model can enhance the students’ knowledge of normal heart anatomy?

☐ Yes ☐ No ☐ Unsure

6) Do you think this model can enhance the students’ knowledge of cardiac pathology?

☐ Yes ☐ No ☐ Unsure

7) Do you think the students will be able to learn the disease quicker using the 3D printed model?

☐ Yes ☐ No ☐ Unsure

**Limitations and Feasibility**

8) Which areas do you think that this model has to be improved for it to suit your teaching needs?

**Overall Satisfaction with the 3D models**

9) How would you rank your overall satisfaction with the 3D model, from 1-10, with 10 being very satisfied.

[ ]
